# Multiple solutions for non-linear radiative mixed convective hybrid nanofluid flow over an exponentially shrinking surface

**DOI:** 10.1038/s41598-023-29892-3

**Published:** 2023-03-01

**Authors:** Mahnoor Sarfraz, Muhammad Yasir, Masood Khan

**Affiliations:** Department of Mathematics, Quaid-I-Azam University, Islamabad, 44000 Pakistan

**Keywords:** Mathematics and computing, Mechanical engineering

## Abstract

Hybrid nanofluids have gained too much attention due to their enhanced thermophysical properties and practical applications. In comparison to conventional nanofluids, their capacity to enhance heat transport is impressive. The simultaneous numerical calculations of hybrid and mono nanofluids across an exponentially shrinking surface in a porous medium are taken into consideration here. The analysis of the thermal energy distribution is carried out by using the convective boundary conditions. Shrinking, permeability, and magnetohydrodynamic controlled the motion of the flow. The objective of this research is to conduct stability analysis and identify the existence of dual solutions in the presence of heat source/sink and nonlinear Roseland thermal radiation. The technique, bvp4c, a collocation method is used to achieve numerical results. It is noted that the energy transport is enhanced immensely due to the presence of a mixture of nanoparticles (hybrid) in comparison to mono nanofluids. The stability analysis shows that the solutions for the upper branch were stable, while the solutions for the lower branch were unstable. Moreover, shrinking parameter contributes significantly to exhibit the dual nature of the solutions. Due to the increment in the heat generation/absorption and temperature ratio, the kinetic energy is inclined, which causes the temperature distribution to rise for both branches. For stable branches, an increase in wall stress values is evident as a result of permeability and stretching of sheet, whereas unstable branches show the opposite trend.

## Introduction

According to the duality principle, optimization problems can be regarded from either the dual problem or the primal problem. The dual problem's resolution establishes a lower constraint for the primal (minimization) problem's resolution. In nanofluids, Subhashini et al*.*^1^ investigated the growth of mixed convection flow at the stagnation point area over an exponentially expanding/constricting sheet. It was expected that the external flow, stretching velocity, and wall temperature would change according to predetermined exponential functions. In the water-based fluid, Bachok et al.^2^ numerically solved the governing equations for an exponentially stretching/shrinking surface for three different types of nanoparticles, including copper, alumina, and titania. Raju et al*.*^3^ performed the heat transfer study for the situations of specified surface temperature (PST) and prescribed heat flux (PHF) across an exponentially extending surface in a porous medium. Raju et al*.*^4^ also proposed dual solutions by contrasting the Casson fluid and viscous fluid results. Recent studies regarding existence of dual solutions are presented in Refs.^5–8^

Choi^9^ introduced the concept of nanofluid. Hybrid nanofluids are made by mixing two or more distinct nanoparticles into the base fluid. The thermal conductivity of the base fluids increases by adding hybrid nanoparticles. The applications of hybrid nanofluids were discussed by Sarkar et al*.*^10^ and Siahchehrehghadikolaei et al*.*^11^. Ghobadi et al*.*^12^ and^13^ developed new thermal conductivity model of nanofluids subject to Ohmic heating. Waini et al*.*^14^ investigated the flow and heat transport of a hybrid nanofluid across an exponentially stretching/shrinking sheet while using combined convection and Joule heating. In order to assess the impact of heat production and absorption on magnetohydrodynamics (MHD) flow toward a bidirectional exponentially stretching/shrinking sheet of hybrid nanofluid, Zainal et al*.*^13^ did a mathematical analysis. Sarfraz and Khan^16^ scrutinized the impact of nanoparticles over a biaxially stretching surface. Nabi et al*.*^17^ observed the increasing energy transport analysis in flat plate containing a mixture of carbon nanotubes and copper oxide nanofluid by using CFD software. Recent studies related to the hybrid nanofluid flow are addressed in Refs.^18–21^. Numerous physicists and mathematicians have investigated the idea of heat transport for stretching surfaces due to their practical applications. Interesting studies on energy transport are addressed in Refs.^22–26^.

Motivated by the above-mentioned research articles, in comparison to conventional nanofluids, hybrid nanofluids ($Al_{2} O_{3}$-$SiO_{2}$ in base fluid $C_{2} H_{6} O_{2}$) are considered as their capacity to enhance heat transport is impressive. The simultaneous numerical calculations of hybrid and mono nanofluids across an exponentially permeable shrinking surface in a porous medium are taken into consideration as well. The analysis of the thermal energy distribution is carried out using the convective boundary conditions. Shrinking parameter and magnetohydrodynamic controlled the motion of the flow. The objective of this research is to conduct stability analysis and identify the existence of dual solutions in the presence of heat source/sink and thermal radiation. The bvp4c collocation method is used to achieve numerical results. The research might be helpful to improve a systems capacity for heat and flow transmission. The fact that nanoparticles have higher thermal and electrical conductivities means that the current findings can also be used for applications involving energy storage and catalytic supports.

## Statement of the problem

**Figure 1:**
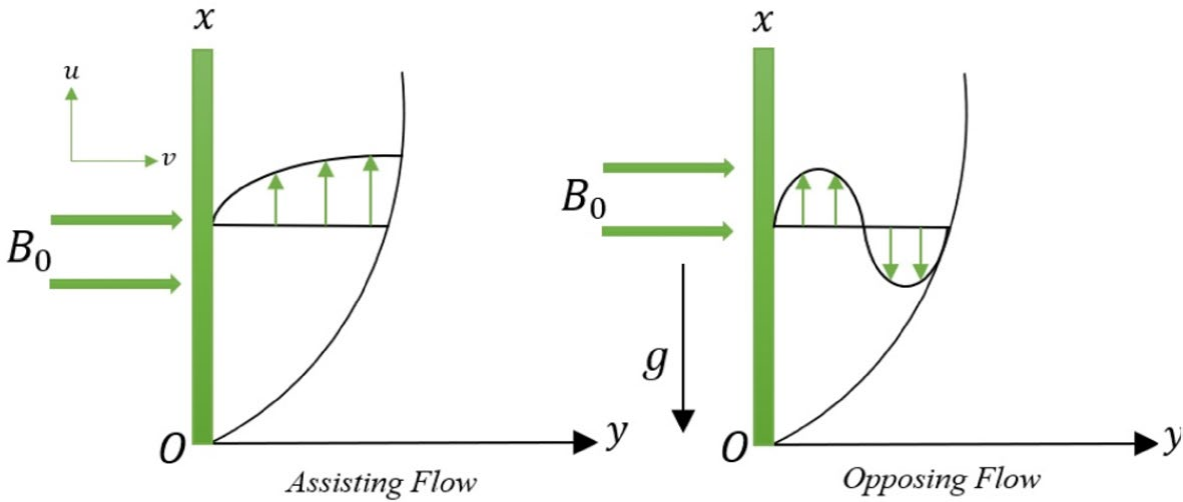
Flow geometry.

We considered the mixture of alumina ($Al_{2} O_{3}$) and silica ($SiO_{2}$) immersed in ethylene glycol ($C_{2} H_{6} O_{2}$) over an exponentially permeable shrinking surface. The convective boundary conditions are used to note the impact of the thermal energy distribution. The simultaneous numerical computations of hybrid nanofluids ($Al_{2} O_{3} - SiO_{2} /C_{2} H_{6} O_{2}$) and mono nanofluids ($Al_{2} O_{3} /C_{2} H_{6} O_{2}$) are noted as well. The flow is governed by shrinking parameter, permeability, and magnetohydrodynamic. The manuscript aims to perform stability analysis and determine the dual solutions in the presence of heat source/sink and thermal radiation. The $x -$ axis is parallel to the plate and $y -$ axis is normal to it along the flow at $y > 0.$ The velocity field is ${\mathbf{V}} = \left[ {u(x,\,y),\,v(x,\,y)} \right]$ with temperature distribution $T.$ The velocity at surface and far field is given as $(u_{w} ,\,u_{e} ),$ respectively. The flow geometry is given in Fig. 1. Table 1 gives the thermophysical properties of nanoparticles ($Al_{2} O_{3}$ and $SiO_{2}$) in base fluid ($C_{2} H_{6} O_{2}$).

**Table 1:** Thermophysical properties of $Al_{2} O_{3}$, $SiO_{2}$, and $C_{2} H_{6} O_{2}$ (see Refs. $\left[ {29,\,30} \right]$).

Physical	Nanoparticles		Base fluid
properties	Alumina	Silica	Ethylene glycol
$c_{p}$	765	745	2430
$k$	46	1.38	0.253
$\beta$	0.85 × 10^−6^	0.55 × 10^−6^	0.57 × 10^−6^
$\rho$	3970	2220	1115
Pr	–	–	2.0363

The model equations for governing equations with hybrid nanomaterials are as follows

1 $$\frac{\partial u}{{\partial x}} + \frac{\partial v}{{\partial y}} = 0,$$

2 $$u\frac{\partial u}{{\partial x}} + v\frac{\partial u}{{\partial y}} = u_{e} \frac{{du_{e} }}{dx} + \frac{{\mu_{hnf} }}{{\rho_{hnf} }}\frac{{\partial^{2} u}}{{\partial y^{2} }} - \frac{{\mu_{hnf} }}{{\rho_{hnf} }}\frac{{\left( {u - u_{e} } \right)}}{{k_{p}^{ * } }} - \frac{{\sigma_{hnf} B_{o}^{2} }}{{\rho_{hnf} }}\left( {u - u_{e} } \right) + \frac{{g\left( {\rho \beta } \right)_{{_{hnf} }} }}{{\rho_{hnf} }}\left( {T - T_{\infty } } \right),$$

3 $$u\frac{\partial T}{{\partial x}} + v\frac{\partial T}{{\partial y}} = \frac{{k_{hnf} }}{{\left( {\rho c_{p} } \right)_{hnf} }}\frac{{\partial^{2} T}}{{\partial y^{2} }} + \frac{{16\sigma^{ * } }}{{3k^{ * } \left( {\rho c_{p} } \right)_{hnf} }}\left\{ {3T^{2} \left( {\frac{\partial T}{{\partial y}}} \right)^{2} + T^{3} \frac{{\partial^{2} T}}{{\partial y^{2} }}} \right\} + \frac{{Q_{o}^{ * } \left( {T - T_{\infty } } \right)}}{{\left( {\rho c_{p} } \right)_{hnf} }},$$

The related boundary conditions are

4 $$\left. {\begin{array}{*{20}l} {u \to u_{w} = be^{{\tfrac{x}{L}}} ,\, \, v = 0,\, \, - k_{hnf} \tfrac{\partial T}{{\partial y}} = h_{f} \left( {T_{w} - T} \right)\,{\text{ at }}y = 0,} \\ {u \to u_{e} = ae^{{\tfrac{x}{L}}} {\text{ and }}T \to T_{\infty } {\text{ as }}y \to \infty ,} \\ \end{array} } \right\},$$

where $b$ is the shrinking rate of the sheet and the choice $a > 0$ manifest the positive constant in the stagnation point flow. The Hamilton-Crosser model $\left[ {7,\,27,\,28} \right]$ for mono nanofluid and hybrid nanofluid are expressed as

5 $$\left. {\begin{array}{*{20}l} {\rho_{nf} = (1 - \phi_{1} )\rho_{f} + \phi_{1} \rho_{{Al_{2} O_{3} }} ,} \\ {\left( {\rho c_{p} } \right)_{nf} = (1 - \phi_{1} )\left( {\rho c_{{_{p} }} } \right)_{f} + \phi_{1} \left( {\rho c_{{_{p} }} } \right)_{{Al_{2} O_{3} }} ,} \\ {\left( {\rho \beta } \right)_{nf} = (1 - \phi_{1} )\left( {\rho \beta } \right)_{f} + \phi_{1} \left( {\rho \beta } \right)_{{Al_{2} O_{3} }} ,} \\ {\tfrac{{k_{nf} }}{{k_{f} }} = \tfrac{{k_{{Al_{2} O_{3} }} + \left( {n - 1} \right)\,k_{f} - \left( {n - 1} \right)\,\left( {k_{f} - k_{{Al_{2} O_{3} }} } \right)\,\phi_{1} }}{{k_{{Al_{2} O_{3} }} + \left( {n - 1} \right)\,k_{f} + \left( {k_{f} - k_{{Al_{2} O_{3} }} } \right)\,\phi_{1} }},} \\ {\mu_{nf} = \tfrac{{\mu_{f} }}{{\left( {1 - \phi_{1} } \right)^{2.5} }},\, \, \nu_{nf} = \tfrac{{\mu_{nf} }}{{\rho_{nf} }},} \\ \end{array} } \right\},$$

and

6 $$\left. {\begin{array}{*{20}l} {\rho_{hnf} = (1 - \phi_{2} )\left( {(1 - \phi_{1} )\rho_{f} + \rho_{{Al_{2} O_{3} }} \phi_{1} } \right) + \phi_{2} \rho_{{SiO_{2} }} ,} \\ {\left( {\rho c_{{_{p} }} } \right)_{hnf} = (1 - \phi_{2} )\left( {(1 - \phi_{1} )\left( {\rho c_{p} } \right)_{f} + \left( {\rho c_{p} } \right)_{{Al_{2} O_{3} }} \phi_{1} } \right) + \phi_{2} \left( {\rho c_{p} } \right)_{{SiO_{2} }} ,} \\ {\left( {\rho \beta } \right)_{hnf} = (1 - \phi_{2} )\left( {(1 - \phi_{1} )\left( {\rho \beta } \right)_{f} + \left( {\rho \beta } \right)_{{Al_{2} O_{3} }} \phi_{1} } \right) + \phi_{2} \left( {\rho \beta } \right)_{{SiO_{2} }} ,} \\ {\tfrac{{k_{hnf} }}{{k_{bf} }} = \tfrac{{k_{{SiO_{2} }} + \left( {n - 1} \right)\,k_{bf} - \left( {n - 1} \right)\,\left( {k_{bf} - k_{{SiO_{2} }} } \right)\,\phi_{2} }}{{k_{{SiO_{2} }} + \left( {n - 1} \right)\,k_{bf} + \left( {k_{bf} - k_{{SiO_{2} }} } \right)\phi_{2} }},\, \, \tfrac{{k_{bf} }}{{k_{f} }} = \tfrac{{k_{{Al_{2} O_{3} }} + \left( {n - 1} \right)\,k_{f} - \left( {n - 1} \right)\,\left( {k_{f} - k_{{Al_{2} O_{3} }} } \right)\,\phi_{1} }}{{k_{{Al_{2} O_{3} }} + \left( {n - 1} \right)\,k_{f} + \left( {k_{f} - k_{{Al_{2} O_{3} }} } \right)\phi_{1} }},} \\ {\mu_{hnf} = \tfrac{{\mu_{f} }}{{\left( {1 - \phi_{1} } \right)^{2.5} \left( {1 - \phi_{2} } \right)^{2.5} }},\, \, \nu_{hnf} = \tfrac{{\mu_{hnf} }}{{\rho_{hnf} }},} \\ \end{array} } \right\},$$

in the above-mentioned expressions, we choose shape factor for the nanoparticles $n = 6,$ as we are interested in the cylindrical shaped nanoparticles.

Assuming the similarity ansatz

7 $$u = ae^{{\tfrac{x}{L}}} f^{^{\prime}} \left( \eta \right),\,\;v = - \sqrt {\frac{{av_{f} }}{2L}} e^{{\tfrac{x}{2L}}} \left[ {f\left( \eta \right) + \eta f^{^{\prime}} \left( \eta \right)} \right],\,\;\theta = \frac{{T - T_{\infty } }}{{T_{w} - T_{\infty } }},\, \, \eta = \sqrt {\frac{a}{{2\nu_{f} L}}} e^{{\tfrac{x}{2L}}} y,$$

the governing Eqs. (2) to (4) are transformed as

8 $$\left. {\begin{array}{*{20}l} {f^{^{\prime\prime\prime}} + {\mathbf{A}}_{1} {\mathbf{A}}_{2} \left( {ff^{^{\prime\prime}} - 2f^{\prime 2} + 2} \right) + {\mathbf{A}}_{1} M\left( {1 - f^{^{\prime}} } \right) + 2k_{p} \left( {1 - f^{^{\prime}} } \right) + 2{\mathbf{A}}_{1} {\mathbf{A}}_{3} \tfrac{{Gr_{x} }}{{{\text{Re}}_{x}^{2} }}\theta = 0,} \\ {f\left( 0 \right) = 0, \, f^{^{\prime}} \left( 0 \right) = \chi ,\, \, f^{^{\prime}} (\infty ) = 1,} \\ \end{array} } \right\},$$

9 $$\left. {\begin{array}{*{20}l} {\theta^{^{\prime\prime}} + \tfrac{4}{3}\tfrac{{R_{d} }}{{{\mathbf{A}}_{4} }}\left[ {\left\{ {1 + \left( {\theta_{w} - 1} \right)\,\theta } \right\}^{3} \theta^{^{\prime\prime}} + 3\left( {\theta_{w} - 1} \right)\,\theta^{\prime 2} \left\{ {1 + \left( {\theta_{w} - 1} \right)\,\theta } \right\}^{2} } \right] + \tfrac{\delta \Pr }{{{\mathbf{A}}_{4} }}\theta + } \\ {\tfrac{{{\mathbf{A}}_{5} }}{{{\mathbf{A}}_{4} }}\Pr \left( {f\theta^{^{\prime}} - f^{^{\prime}} \theta } \right) = 0,\, \, {\mathbf{A}}_{5} \theta^{^{\prime}} \left( 0 \right) = \gamma \left\{ {1 - \theta \left( 0 \right)} \right\},\, \, \theta (\infty ) = 0.} \\ \end{array} } \right\},$$

The dimensionless parameters are

10 $$\left. {\begin{array}{*{20}l} {\chi = \tfrac{b}{a},\, \, M = \tfrac{{\sigma_{f} B_{o}^{2} }}{{a?_{f} }},\, \, Gr_{x} = \tfrac{{g\beta_{{_{f} }} (T_{w} - T_{\infty } )L^{3} }}{{v_{f}^{2} }},\, \, Re\left( { = \tfrac{{u_{w} x}}{{v_{f} }}} \right),\,\gamma \left( { = \tfrac{{h_{f} }}{{k_{0} }}\sqrt {\tfrac{\nu }{a}} } \right),} \\ {k_{p} = \tfrac{{v_{f} L}}{{ak_{p}^{ * } }},\, \, \Pr = \tfrac{{v_{f} }}{{\alpha_{f} }},\,\theta_{w} = \left( {\tfrac{{T_{w} }}{{T_{\infty } }}} \right) > 1,\,R_{d} = \tfrac{{4\sigma^{ * } T_{\infty }^{3} }}{{k^{ * } k_{f} }},\, \, \delta \left( { = \tfrac{{Q_{o} }}{{a\left( {\rho c_{p} } \right)_{f} }}} \right),} \\ {{\mathbf{A}}_{1} = \tfrac{{\mu_{hnf} }}{{\mu_{f} }},\, \, {\mathbf{A}}_{2} = \tfrac{{\rho_{hnf} }}{{\rho_{f} }},\, \, {\mathbf{A}}_{3} = \tfrac{{\left( {\rho \beta } \right)_{hnf} }}{{\left( {\rho \beta } \right)_{f} }},\, \, {\mathbf{A}}_{4} = \tfrac{{\left( {\rho c_{{_{p} }} } \right)_{hnf} }}{{\left( {\rho c_{{_{p} }} } \right)_{f} }},\, \, {\mathbf{A}}_{5} = \tfrac{{k_{hnf} }}{{k_{f} }}.} \\ \end{array} } \right\},$$

The quantities of engineering interest include skin friction coefficient $C_{f}$ and rate of heat transfer $Nu_{x}$, which are expressed as

11 $$\left. {\begin{array}{*{20}l} {\begin{array}{*{20}l} {C_{f} = \tfrac{{\tau_{w} }}{{\rho_{f} u_{w}^{2} }},\,{\text{ where }}\tau_{w} = \mu_{hnf} \left. {\left( {\tfrac{\partial u}{{\partial y}}} \right)} \right|_{y = 0} ,} \\ \end{array} } \\ {Re^{{\tfrac{1}{2}}} C_{{f_{x} }} = {\mathbf{A}}_{1} f^{^{\prime\prime}} \left( 0 \right),} \\ \end{array} } \right\},$$

12 $$\left. {\begin{array}{*{20}l} {\begin{array}{*{20}l} {Nu_{x} = \tfrac{{xq_{w} }}{{k_{f} (T_{w} - T_{\infty } )}}{\text{ where }}q_{w} = - k_{hnf} \left. {\left( {\tfrac{\partial T}{{\partial y}}} \right)} \right|_{y = 0} + \left. {\tfrac{{16\sigma^{ * } T^{3} }}{{3k^{ * } \left( {\rho c_{p} } \right)_{hnf} }}\left( {\tfrac{\partial T}{{\partial y}}} \right)} \right|_{y = 0} ,} \\ \end{array} } \\ {Re^{{ - \tfrac{1}{2}}} Nu_{x} = - {\mathbf{A}}_{5} \left[ {1 + \tfrac{4}{3}\tfrac{{R_{d} }}{{{\mathbf{A}}_{5} }}\left( {1 + \left( {\theta_{w} - 1} \right)\,\theta \left( 0 \right)} \right)^{3} } \right]\,\theta^{^{\prime}} \left( 0 \right).} \\ \end{array} } \right\}.$$

### Solution approach

In this section, we discuss the procedure for numerical solution for the dimensionless ordinary differential equations $\;\left( {8){\text{ and }}(9} \right).$ These equations are solved numerically by using *bvp4c* solver in Matlab. In this method, the use of initial guess is required in order to solve the BVPs. It works on a finite difference method. This tool uses the Lobatto IIIa formula to produce ${\mathbf{C}}^{1}$ continuous solutions, remotely monitor error tolerances, and provide solutions to the issue. We use the following transformations to convert the higher order differential equations into first order differential equations.

13 $$f = \varepsilon_{1} ,\,\;f^{^{\prime}} = \varepsilon_{2} ,\,\;f^{^{\prime\prime}} = \varepsilon_{3} ,\,\;f^{^{\prime\prime\prime}} = \varepsilon \varepsilon_{1} ,\,\;\theta = \varepsilon_{4} ,\,\;\theta^{^{\prime}} = \varepsilon_{5} ,\,\;\theta^{^{\prime\prime}} = \varepsilon \varepsilon_{2} ,$$

14 $$\varepsilon \varepsilon_{1} = - {\mathbf{A}}_{1} {\mathbf{A}}_{2} \left( {\varepsilon_{1} \varepsilon_{3} - 2\varepsilon_{1}^{2} + 2} \right) - {\mathbf{A}}_{1} M\left( {1 - \varepsilon_{2} } \right) - 2k_{p} \left( {1 - \varepsilon_{2} } \right) - 2{\mathbf{A}}_{1} {\mathbf{A}}_{3} \frac{{Gr_{x} }}{{Re_{x}^{2} }}\varepsilon_{4} ,$$

15 $$\varepsilon\varepsilon_2= \tfrac{ \mathbf{A_5}Pr(\varepsilon_2\varepsilon_4-\varepsilon_1\varepsilon_5)-\tfrac{4R_d}{\mathbf{A_4}}(\theta_w-1)\varepsilon_5^2\left\{{1+(\theta_w-1)\varepsilon_4}\right\}^2-\tfrac {\delta Pr \varepsilon_4} {\mathbf{A_4}}}{\mathbf{A_5}+\tfrac{4R_d}{3\mathbf{A_4}}\left\{{1+(\theta_w-1)\epsilon_4}\right\}^3},$$

with

16 $$\varepsilon_{1} (0) = S,\,\;\varepsilon_{2} (0) = \chi ,\,\;\varepsilon_{2} (\infty ) = 1,\,\;{\mathbf{A}}_{5} \varepsilon_{5} \left( 0 \right) = \gamma \left\{ {1 - \varepsilon_{4} \left( 0 \right)} \right\},\,\;\varepsilon_{4} (\infty ) = 0.$$

## Results interpretation

This section discusses the results obtained for the coefficient of skin friction, Nusselt number, temperature, and velocity profiles. The mathematical technique, *bvp4c*, a collocation method, is used to compute the dual solutions of the problem. In this technique, the higher-order differential equations are converted into a system of first-order differential equations. An initial guess is provided, and the error and mesh points are incorporated within the program.

**Figure 2:**
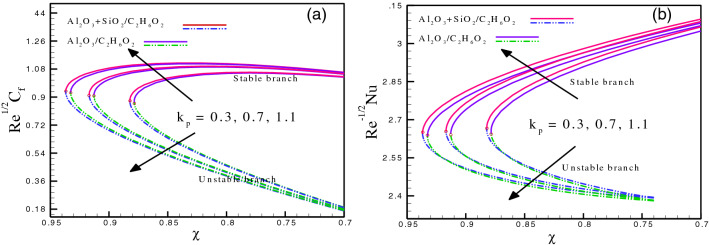
(**a**) and (**b**): Variation of $Re^{{\tfrac{1}{2}}} C_{f}$ and $Re^{{ - \tfrac{1}{2}}} Nu$ for different values of $k_{p} .$

**Figure 3:**
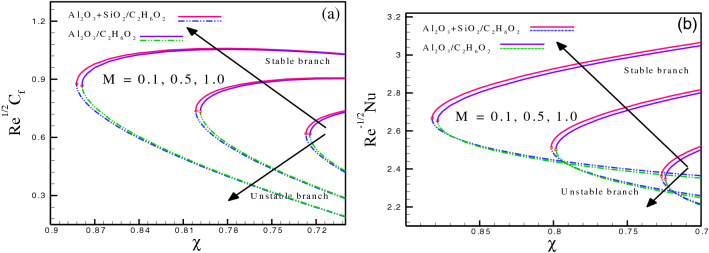
(**a**) and (**b**): Variation of $Re^{{\tfrac{1}{2}}} C_{f}$ and $Re^{{ - \tfrac{1}{2}}} Nu$ for different values of $M.$

**Figure 4:**
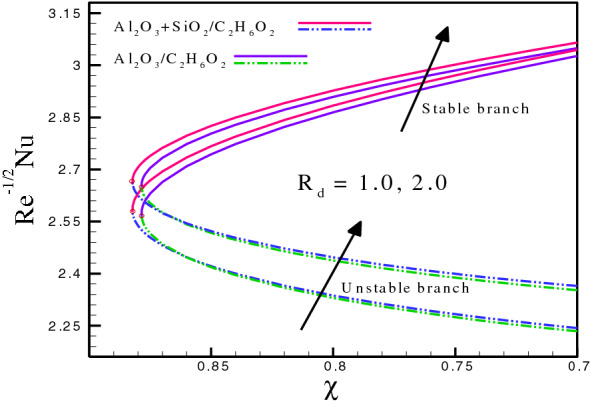
Variation of $Re^{{ - \tfrac{1}{2}}} Nu$ for different values of $R_{d} .$

Figure 2a and b provide the impact of the permeability parameter on the coefficient of skin friction and Nusselt number. The variation of $k_{p}$ ranges from 0.3 to 1.1 for both hybrid nanofluid and mono nanofluid. Figure 2a gives the critical points for hybrid nanofluid case ($Al_{2} O_{3} - SiO_{2} /C_{2} H_{6} O_{2}$) are $\chi_{c} = - 0.936856,$ $- 0.916721,$ $- 0.88217$ at $k_{p} = 0.3,\,0.7,\,1.1,$ respectively, for $Re^{{\tfrac{1}{2}}} C_{f} .$ For mono nanofluid case ($Al_{2} O_{3} /C_{2} H_{6} O_{2}$), $\chi_{c} = - 0.932506,$ $- 0.91262,$ $- 0.878317$ at $k_{p} = 0.3,\,0.7,\,1.1,$ respectively. These values are visible at $\chi < 0,$ which indicates that this parameter causes the dual behavior of the solution. The increment in values of wall stress is observed due to variation of permeability parameter for the stable branch, while contrasting behavior is seen for the unstable branch. Figure 2b illustrates the behavior of $Re^{{\tfrac{ - 1}{2}}} Nu$ for both hybrid and mono nanofluid cases. The critical points for hybrid nanofluid case ($Al_{2} O_{3} - SiO_{2} /C_{2} H_{6} O_{2}$) are $\chi_{c} = - 0.936623,$ $- 0.916855,$ $- 0.882026$ at $k_{p} = 0.3,\,0.7,\,1.1,$ respectively, for $Re^{{\tfrac{ - 1}{2}}} Nu.$ For mono nanofluid case ($Al_{2} O_{3} /C_{2} H_{6} O_{2}$), $\chi_{c} = - 0.932588,$ $- 0.912821,$ $- 0.87853$ at $k_{p} = 0.3,\,0.7,\,1.1,$ respectively. The comparison of hybrid nanofluids with mono nanofluids shows that the energy transport is enhanced due to the presence of a mixture of nanoparticles in ethylene glycol. The enhancement in heat distribution is due to permeability as well. Therefore, due to the increment in permeability parameter, the heat transport magnifies for the stable branch, whereas an opposite trend is visible for the unstable branch. Figure 3a and b depict the impact of magnetic parameter on $Re^{{\tfrac{1}{2}}} C_{f}$ and $Re^{{\tfrac{ - 1}{2}}} Nu.$ The variation of $M$ ranges from $3.0$ to $4.0.$ Figure 3a presents the effect of $M$ on $Re^{{\tfrac{1}{2}}} C_{f}$ for both hybrid and mono nanofluids. Magnetic field generates an opposing force, known as Lorentz force, which increases the fluid friction. For stable branches, an increase in wall stress values is evident as a result of magnetic parameter variation, whereas unstable branches exhibit the opposite trend. The critical values for hybrid nanofluids ($Al_{2} O_{3} - SiO_{2} /C_{2} H_{6} O_{2}$) are $\chi_{c} = - 0.882151,$ $- 0.801498,$ $- 0.726856$ at $M = 0.1,\,0.5,\,1.0,$ respectively. However, for mono nanofluids ($Al_{2} O_{3} /C_{2} H_{6} O_{2}$) $\chi_{c} = - 0.87835,$ $- 0.798415,$ $- 0.724203$ at $M = 0.1,\,0.5,\,1.0,$ respectively. For stable branches, an increase in wall stress values is evident as a result of magnetic parameter variation, whereas unstable branches exhibit the opposite trend. For $Re^{{\tfrac{ - 1}{2}}} Nu,$ the critical values for $Al_{2} O_{3} - SiO_{2} /C_{2} H_{6} O_{2}$ are $\chi_{c} = - 0.882215,$ $- 0.801433,$ $- 0.726649$ at $S = 3.0,\,3.5,\,4.0,$ respectively. However, for $Al_{2} O_{3} /C_{2} H_{6} O_{2}$ $\chi_{c} = - 0.878475,$ $- 0.797955,$ $- 0.724214$ at $S = 3.0,\,3.5,\,4.0,$ respectively. It can be seen through a comparison of hybrid and mono nanofluids that the inclusion of a variety of nanoparticles in ethylene glycol improves energy transmission. Magnetic field also contributes to the improvement in heat dispersion. As a result, the heat transfer for the stable branch increases due to the increase in $M$ , but the unstable branch shows an opposite pattern. Figure 4 highlight the influence of radiation parameter on $Re^{{\tfrac{ - 1}{2}}} Nu.$ The variation of $R_{d}$ is from $1.0$ to $2.0.$ Figure 4 describes the effect of $R_{d}$ on $Re^{{\tfrac{ - 1}{2}}} Nu$ for both hybrid and mono nanofluids. The critical values for hybrid nanofluids $(Al_{2} O_{3} - SiO_{2} /C_{2} H_{6} O_{2} )$ are $\chi_{c} = - 0.882181,$ $- 0.882181$ at $R_{d} = 1.0,\,2.0,$ respectively. However, for mono nanofluids $\;(Al_{2} O_{3} /C_{2} H_{6} O_{2} )$ $\chi_{c} = - 0.878543,$ $- 0.878543$ at $R_{d} = 1.0,\,3.0,$ respectively. The values at these points for both cases are exactly the same. The variation shows that both branches (stable and unstable) cause the heat transfer rate to boost significantly.

**Figure 5:**
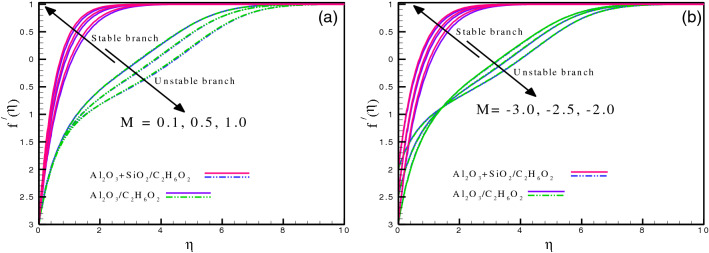
(**a**) and (**b**): Variation of $f^{^{\prime}} \left( \eta \right)$ for different values of $M\;$ and $\chi .$

**Figs. 6:**
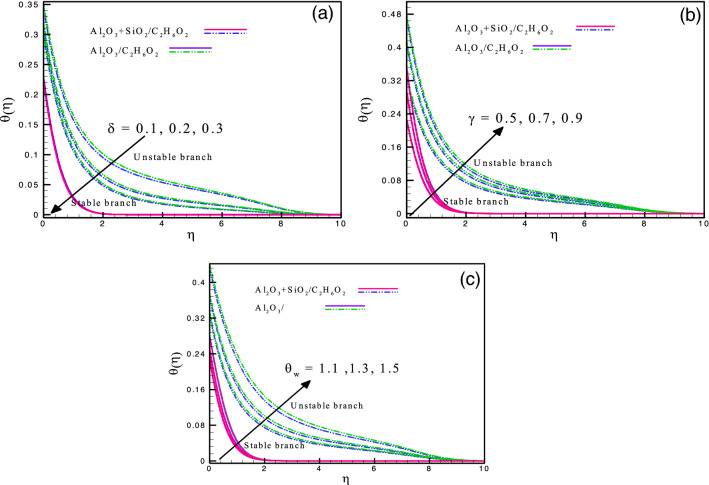
(**a**)–(**c**): Variation of $\theta \left( \eta \right)$ for different values of $\delta ,$ $\gamma ,$ and $\theta_{w} .$

Figure 5a and b demonstrate the impression of magnetic parameter and shrinking parameter on $f^{\prime}(\eta ),$ respectively. The impression of $M$ is noted on the velocity field and is seen to be increasing for the case of the stable branch. The values of $M$ range from $0.1$ to $1.0.$ On the other hand, the unstable branch shows the opposite trend for both cases of nanofluid, *i.e.*, hybrid and mono ($Al_{2} O_{3} - SiO_{2} /C_{2} H_{6} O_{2}$ and $Al_{2} O_{3} /C_{2} H_{6} O_{2}$). The decreasing behavior of the velocity field for the stable branch is due to Lorentz force induced by magnetic field effect. A boundary layer structure with high suction velocity may be seen in the flow close to the plate. Similarly, Fig. 5b exhibits that the velocity field is an increasing function of the shrinking parameter. The stretching causes the fluid motion to augment which in response boosts the velocity field significantly. Figure 6a–c illustrate the impact of heat source/sink parameter, Biot number, and temperature ratio on $\theta \left( \eta \right),$ respectively. It is observed that the thermal energy transfer effects are increased due to an increment in the energy absorption/generation, as given in Fig. 6a. Physically, the heat source/sink gives a passive heat exchanger that transfers energy without changing temperature (ideally). Heat generation $(\delta < 0)$ makes the fluid warmer and thickens the boundary layer; whilst heat absorption $(\delta < 0)$ has a reverse impact. The Biot number $\gamma$ is the ratio of the body's internal conductive resistance (unlike the Nusselt number) to its outward convective resistance at its surface. It depicts the relationship between convective resistance at an object's surface instead of fluids and interior conductive resistance. The analysis of lumped systems uses it as a criterion. An object's internal resistance is higher than its outward resistance if $\gamma$ is larger. The Biot number's lower value denotes how much less external resistance an object's conductive resistance is in comparison to that of the environment. Due to an increase in $\gamma ,$ the surface heat resistance declines which dominate the convection mechanism resulting in a higher temperature field. Figure 6b shows that enhancing $\gamma$ causes the thermal transport to incline as well. Figure 6c discusses the impact of temperature ratio on thermal energy transport. It is the ratio between the liquid's ambient temperature and the temperature of the hybrid nanofluid at the sheet surface. It is seen that for both branches the heat distribution is an increasing function of $\theta_{w} .$ As heat is provided, the system's temperature is elevated, which is a response to the random motion of the molecules (including nanoparticles). Therefore, the heat transport is enhanced and consequently raises the system's kinetic energy and temperature field.

## Stability analysis

**Figure 7:**
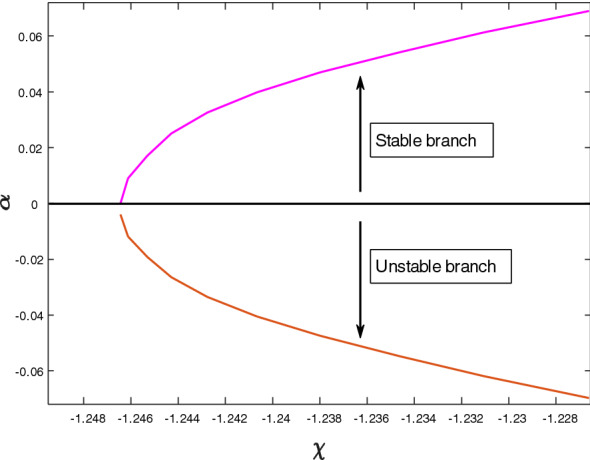
The smallest eigenvalues $\alpha$ for various values of $\chi$.

The stability analysis of a current study is conducted when the governing system admits multiple branch solutions. To validate the actual solution, the mathematician must determine every solution that could result from the governing boundary layer problems. With the help of the following previous works of literature $\left[ {31 - 36} \right]$, the smallest eigenvalues $\alpha$ against $\chi$ are plotted in Fig. 7. The physical phenomenon in this figure states that positive value $\alpha$ of conforms to an initial deterioration of disturbance signifies that the flow is in a stable mode. Meanwhile, the flow is in an unstable condition as $\tau \to \infty$ indicated by the negative value of $\alpha$, which is associated with the early increase of disturbance. It is worth noting that when $\alpha$ tends to zero as $\chi$ approaches the crucial value, $\chi_{c} = - 1.2431$ for both the stable and unstable branches. This behavior indicates that the solutions split at the critical values.

### Validation of results

The numerical results are verified by comparing them to the numerical results of the current investigation, which were reported in Ref.^36^ for various values of the shrinking parameter and are shown in Table 2. There is confidence in the accuracy of the current process because the findings are in good accord.

**Table 2:** The comparison of $Re^{{\tfrac{1}{2}}} C_{f}$ for regular fluid $\left( {\phi_{1} = \phi_{2} = 0} \right)$ with shrinking parameter $\chi$.

	Hafeez et al.^36^		Present results	
$\chi$	Upper branch	Lower branch	Upper branch	Lower branch
− 0.25	1.4022331	–	1.402915	–
− 0.50	1.4958565	–	1.494583	–
− 0.75	1.4893224	–	1.483975	–
− 1.00	1.3281191	–	1.320169	–
− 1.15	1.0823058	0.1160042	1.089714	0.117017
− 1.20	0.9320782	0.2336751	0.932146	0.234294

## Conclusion

The article considered the simultaneous numerical computations of hybrid nanofluids $(Al_{2} O_{3} - SiO_{2} /C_{2} H_{6} O_{2} )$ and mono nanofluids $(Al_{2} O_{3} /C_{2} H_{6} O_{2} )$ over an exponentially permeable stretching/shrinking surface over a porous medium. The convective boundary conditions were used to note the impact of the thermal energy distribution. The flow was induced by shrinking parameter and magnetohydrodynamic. The manuscript aimed to perform stability analysis and determine the dual solutions in the presence of heat source/sink and thermal radiation. The numerical outcomes were attained through a collocation method namely, bvp4c. The present work can be extended for the energy transport examination of non-Newtonian fluids. Also, the thermodynamic irreversibility analysis can be a good approach to study in future. The key points are given as:

- The energy transport was enhanced immensely due to the presence of a mixture of nanoparticles (hybrid) in comparison to mono nanofluids.
- Stability analysis showed that the solutions for upper branch were stable, while the solutions for lower branch were unstable.
- The critical values obtained due to variation of thermal radiation parameter were identical for hybrid and mono nanofluids.
- Temperature of the system was an increasing function of heat source/sink, Biot number, and temperature ratio for both branches.
- The fluid motion was augmented due to shrinking parameter and magnetic field for hybrid nanofluids and mono nanofluids.
- Shrinking parameter contributed significantly to exhibit the dual nature of the solutions.
- For stable branches, an increase in wall stress values was evident as a result of permeability parameter, magnetic parameter, and stretching parameter, whereas unstable branches showed the opposite trend.
- The heat transport rate was boosted due to the variation of radiation parameter for upper and lower branches.
- Due to the increment in permeability and shrinking, the energy transmission magnified for the stable branch, whereas an opposite trend was visible for the unstable branch.

## Data Availability

No datasets were generated or analysed during the current study.

## Abbreviations

$$u,\,v$$: Velocity components [$${\text{ms}}^{ - 1}$$]
$$T_{\infty }$$: Ambient temperature [K]
$$M$$: Magnetic parameter
$$\Pr$$: Prandtl number
$$T$$: Fluid temperature [K]
$$R_{d}$$: Thermal radiation
$\chi$: Stretching/shrinking parameter
$\delta$: Heat source/sink parameter

## For hybrid nanofluid

$$\rho_{hnf}$$: Density
$$\mu_{hnf}$$: Dynamic viscosity
$$\nu_{hnf}$$: Kinematic viscosity
$\phi_{2}$: SiO_2_ volume fraction
$$\alpha_{hnf}$$: Thermal diffusivity
$\left( {c_{p} } \right)_{hnf}$: Specific heat capacity
$$k_{hnf}$$: Thermal conductivity
$\beta_{hnf}$: Thermal expansion coefficient

## For nanofluid

$$\rho_{nf}$$: Density
$$\nu_{nf}$$: Kinematic viscosity
$\mu_{nf}$: Dynamic viscosity [kgm $^{ - 1}$ s $^{ - 1}$]
$\beta_{nf}$: Thermal expansion coefficient
$$k_{nf}$$: Thermal conductivity
$$\alpha_{nf}$$: Thermal diffusivity
$\left( {c_{p} } \right)_{nf}$: Specific heat capacity
$\phi_{1}$: Al_2_O_3_ volume fraction

## For base fluid

$$\rho_{f}$$: Density
$$\nu_{f}$$: Kinematic viscosity
$$\mu_{f}$$: Dynamic viscosity
$\beta_{f}$: Thermal expansion coefficient
$$k_{f}$$: Thermal conductivity
$$\alpha_{f}$$: Thermal diffusivity
$\left( {c_{p} } \right)_{f}$: Specific heat capacity
